# Design and Biological Profiling of a Drug-like Chloropyridine Diamine as a Dual Antioxidant–Antimicrobial Lead: In Vitro Evaluation and In Silico Multi-Target Studies

**DOI:** 10.3390/ijms27062777

**Published:** 2026-03-19

**Authors:** Oussama Merzouki, Elhachmia Ech-chihbi, Nadia Arrousse, El Houssine Mabrouk, Mohamed Hefnawy, Yasmine Fernine, Manal El-Gendy, Mustapha Taleb

**Affiliations:** 1 Laboratory of Engineering Electrochemistry, Modeling, and Environment, Department of Chemistry, Faculty of Sciences Dhar Mahraz, Sidi Mohamed Ben Abdellah University, Fez 30000, Morocco; 2 Laboratory of Physics and Chemistry of Inorganic and Organic Materials, Higher Normal School, Mohammed V University, Rabat 30050, Morocco; 3 School of Science and Engineering, Al Akhawayn University in Ifrane, Hassan II Avenue, Ifrane 53000, Morocco; 4 Laboratory of Materials Engineering for the Environment and Natural Ressources, Faculty of Sciences and Technics, University of Moulay Ismail, Meknes, B.P 509, Boutalamine, Errachidia 52000, Morocco; 5 Department of Pharmaceutical Chemistry, College of Pharmacy, King Saud University, Riyadh 11451, Saudi Arabia; 6 CICECO—Aveiro Institute of Materials and Department of Chemistry, University of Aveiro, 3810-193 Aveiro, Portugal

**Keywords:** synthesis, chloropyridine derivative, biological activities, molecular docking, ADMET

## Abstract

Bacterial and fungal infections, together with oxidative stress-mediated damage, remain major challenges in human health and in the protection of materials, highlighting the need for new multifunctional molecules that combine antioxidant and antimicrobial properties. In this context, a new chloropyridine-based derivative, N4,N4-bis((6-chloropyridin-3-yl)methyl)-N1,N1-diethylpentane-1,4-diamine (AMZ), was synthesized via a simple, catalyst-free N-alkylation of N1,N1-diethylpentane-1,4-diamine with 2-chloro-4-(chloromethyl)pyridine in acetonitrile at 55 °C, affording a 62% yield. The structure of AMZ was confirmed by melting point determination, ^1^H and ^13^C NMR spectroscopy, and EI–MS analysis. Its antioxidant activity was evaluated using DPPH and FRAP assays with BHT as a reference standard, while antibacterial and antifungal activities were assessed via disk diffusion and microdilution methods to determine inhibition zones and MIC/MBC values. In silico investigations included drug-likeness and ADMET predictions, as well as molecular docking on catalase (PDB: 2CAG) and fungal CYP51 (PDB: 1EA1). AMZ exhibited dose-dependent radical scavenging in the DPPH assay, reaching 76.88 ± 3.20% inhibition at 1000 µg/mL, with an EC_50_ of 26.03 ± 0.21 µg/mL, close to that of BHT (23.65 ± 0.22 µg/mL). In the FRAP assay, AMZ showed a higher reducing power than BHT at a low concentration (OD_50_ µg/mL 0.177 ± 0.023 vs. 0.134 ± 0.017), although its FRAP EC_50_ was higher (700.48 ± 22.54 vs. 400.16 ± 8.67 µg/mL). AMZ displayed broad-spectrum antimicrobial activity against Gram-positive and Gram-negative bacteria and fungi, with particularly strong effects on *Bacillus subtilis* (44.5 ± 0.5 mm; MIC/MBC 0.008 mg/mL) and *Aspergillus niger* (30 mm; MIC/MBC 0.030 mg/mL), in some cases comparable or superior to streptomycin and fluconazole. In silico analysis indicated that AMZ fulfilled major drug-likeness rules, showed high predicted intestinal absorption (91.14%), and was classified as non-AMES toxic, while docking predicted favorable binding to catalase and CYP51, in agreement with the experimental antioxidant and antifungal activities. These findings highlight the potential of AMZ as a multi-target pyridine-based lead compound that warrants further structural optimization and in vivo evaluation for applications in oxidative-stress-related and infectious conditions.

## 1. Introduction

Bacterial infections, fungal overgrowth, and oxidative stress are major challenges across diverse areas such as science, medicine, and industry. Bacterial contamination, beyond its role as a cause of many infections and diseases, represents a significant threat in clinical environments due to the emergence of antibiotic-resistant strains [1,2]. In addition, invasive infections are elicited by filamentous fungi, which can result in fatal consequences for immunocompromised patients and remain responsible for a major portion of fungal-related deaths, as well as an ever-present threat to agriculture, food storage and pharmaceuticals, causing spoilage and quality diminution [3,4]. Such challenges are further aggravated by the occurrence of oxidative stress, which results from an imbalance between the generation of reactive oxygen species (ROS) and the detoxification capacity of the body. It is associated with cell injury, senescence, and chronic diseases such as cancer, cardiovascular and neurodegenerative disease [5]. Moreover, oxidative stress promotes the release of metal ions and accelerates degradation processes that lead to corrosion of materials [6], which represents a major economic burden in construction, transportation and energy infrastructures [7]. Solving the issues posed by these interconnected challenges involves developing multi-active compounds that are active against bacterial and fungal contaminants, as well as oxidative stress, a destructive mechanism in biological and material systems.

Pyridine derivatives have been established as drug candidates due to their wide-ranging biological and chemical activities [8,9,10,11,12]. These compounds are known to interfere with the microbial cell wall or enzymes required for microbial metabolism and have been widely studied for their antimicrobial activity. Several of them show potent antibacterial and antifungal activities [11,13,14]. Besides their antibacterial effect, pyridine derivatives have also exhibited strong antioxidant activity. These compounds can scavenge free radicals and/or block oxidative mechanisms to reduce the cellular and material damage produced by ROS [15]. Among pyridine-based scaffolds, chloropyridine was selected as a relevant heteroaromatic motif because it combines the pharmacophoric features of a nitrogen-containing aromatic ring with the electronic and lipophilic contribution of a chlorine substituent. In parallel, N1,N1-diethylpentane-1,4-diamine was selected as a drug-like aminoalkyl platform suitable for stepwise N-alkylation. In our previous work on the same scaffold, a mono-chloropyridine analogue was already accessible [16]. Therefore, in the present study, we deliberately targeted the bis-chloropyridinylmethyl derivative AMZ as the logical structural extension of that mono analogue. This choice made it possible to evaluate the effect of duplicating the same chloropyridine fragment on a constant diamine backbone, thereby isolating the influence of the substitution degree (mono vs. bis) without simultaneously changing the pyridine-ring substitution pattern. Accordingly, the present work should be regarded as a focused proof-of-concept study rather than a full structure–activity relationship campaign.

Because microbial infection and oxidative stress are frequently interconnected, the development of a molecule combining antimicrobial and antioxidant properties may be therapeutically advantageous. In infectious conditions, microbial proliferation can trigger reactive oxygen species production and inflammatory damage, while oxidative stress may further aggravate tissue injury and delay recovery. Therefore, a dual-function scaffold may provide complementary benefits by reducing pathogen burden while also limiting collateral oxidative damage. The aim of this work is to synthesize a new pyridine-based compound and investigate its multiple effects on antioxidant and antifungal activity. Both experimental and computational (including DFT calculations and pharmacokinetic profiling) approaches are used. Overall, this integrative approach presents a comprehensive evaluation of the potential applications of the compound and provides evidence supporting it as an agent with broad function for enumerating multiple health conditions. This article aims to unify experimental synthesis and computational analysis as a single entity, and it hopes to provide valuable input for the development of pyridine-based solutions to today’s scientific challenges.

## 2. Results and Discussion

### 2.1. General Method of the Synthesis of N4,N4-Bis((6-chloropyridin-3-yl)methyl)-N1,N1-diethylpentane-1,4-diamine (AMZ)

**Scheme 1:**
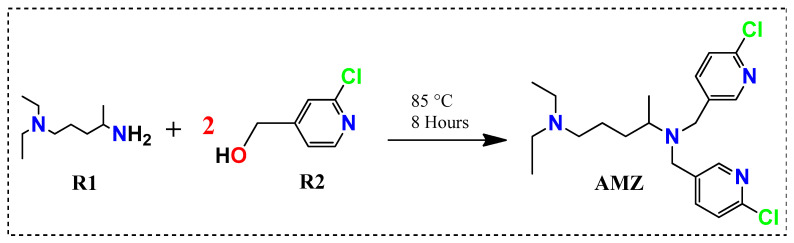
The synthesis route of AMZ compound.

The synthesis of AMZ was achieved through a simple catalyst-free N-alkylation of N1,N1-diethylpentane-1,4-diamine (3 mL, 15.19 mmol) with 2-chloro-5-(chloromethyl)pyridine (4.89 g, 30.38 mmol) in acetonitrile (20 mL) at 55 °C for 8 h (Scheme 1). After evaporation of the solvent, the residue was washed with diethyl ether to afford AMZ as a light brown solid (3.84 g, 62%). Mechanistically, the reaction can be rationalized as a sequential SN2-type alkylation of the diamine by the chloromethyl pyridine electrophile, favored by the polar aprotic medium. The isolation of the bis-substituted product is consistent with stepwise alkylation of the same diamine scaffold and was intentionally targeted here as the logical extension of the previously obtained mono analogue on this platform [16]. The proposed structure of AMZ was supported by corrected ^1^H (Figure S1) and ^13^C (Figure S2) NMR data together with exact-mass mass spectrometry (Figure S3). In particular, the residual CDCl3 signal at δ 7.24–7.26 ppm was excluded from the proton assignment, whereas the benzylic NCH_2_-pyridyl protons were assigned at δ 3.69–3.78 ppm. The 13 distinct carbon resonances observed in the ^13^C NMR spectrum are consistent with the expected symmetry of AMZ. Suitable single crystals were not available from the isolated material; therefore, no single-crystal X-ray diffraction study is reported in the present work.

This methodology highlights a straightforward and reproducible approach to AMZ synthesis under mild and catalyst-free N-Alkylation. While the yield of 62% is moderate, it reflects the simplicity and practicality of the reaction, which avoids toxic reagents and harsh conditions. The successful characterization of AMZ provides a foundation for further pharmacological exploration, demonstrating the potential of this sustainable synthesis approach in drug development.

### 2.2. Global Reactivity Analysis Using the DFT Method

**Figure 1:**
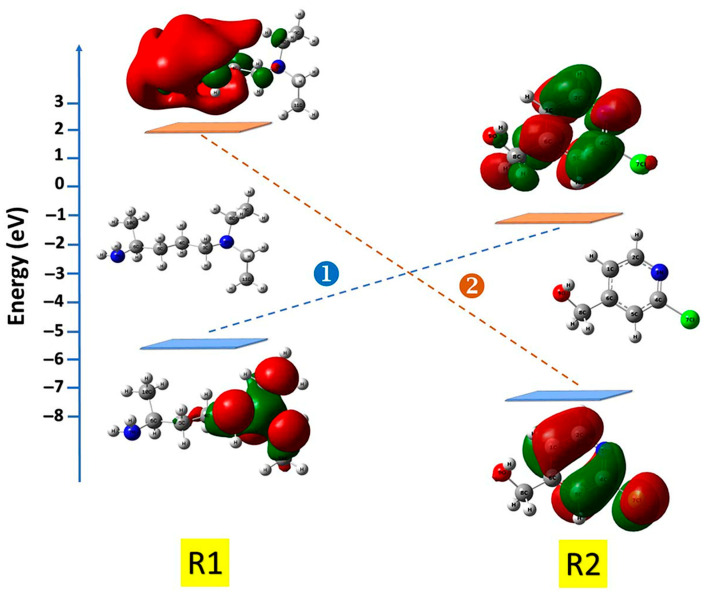
Energy diagram of the Highest Occupied Molecular Orbitals (HOMOs) and Lowest Unoccupied Molecular Orbitals (LUMOs) for the compounds R1 and R2.

Figure 1 presents the energy diagram of the frontier molecular orbitals (FMOs), i.e., the Highest Occupied Molecular Orbitals (HOMOs) and Lowest Unoccupied Molecular Orbitals (LUMOs), for R1 and R2. The HOMO of R1 is mainly localized on the carbon atom bound to the metal and on the adjacent π-system, confirming that this region is the principal electron-donor site and can participate as the nucleophilic partner [17]. In contrast, the LUMO of R2 is predominantly distributed over the aromatic ring and especially on the ipso carbon bearing the chlorine substituent, making this position the most favorable electron-acceptor site for nucleophilic attack. The complementary spatial distribution between the HOMO of R1 and the LUMO of R2, combined with their relatively small energy separation (pathway ① in Figure 1), supports a charge transfer from R1 to R2 and thus the feasibility of the proposed C–C bond formation. The alternative interaction involving the HOMO of R2 and the LUMO of R1 (pathway ②) is less favorable due to poorer overlap and a larger energy gap and is therefore not expected to compete significantly. Overall, the MEP analysis and FMO description are consistent with the predicted direction of electron flow and the regioselectivity of the designed organic synthesis.

**Figure 2:**
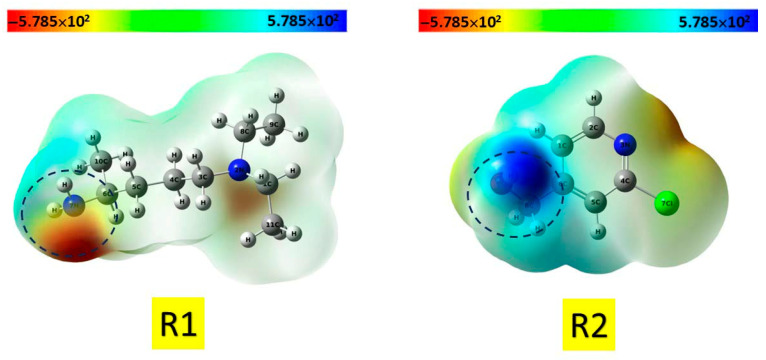
Optimized geometries and molecular electrostatic potential (MEP) maps of compounds R1 and R2.

The molecular electrostatic potential (MEP) maps of reagents R1 and R2 are shown in Figure 2. The MEP surfaces describe the three-dimensional distribution of the electrostatic potential around each molecule, with a color scale ranging from negative (red) to positive (blue) values. Red regions correspond to electron-rich domains, which are expected to behave as potential nucleophilic sites, whereas blue regions indicate electron-deficient areas with electrophilic character. For R1, the most negative potentials are mainly localized on the fragment bearing the metal center and along the conjugated chain (dashed contour), indicating that this part of the molecule can act as an electron-donating center in the reaction. In R2, the potential is relatively positive around the aromatic carbon bonded to the chlorine atom, while the N-heterocycle displays more negative values, suggesting that the aryl chloride moiety is the preferred electrophilic site in the envisaged transformation.

Table 1 gathers the electronic FMO energies and global reactivity descriptors of reagents R1 and R2. The HOMO and LUMO energies show that R1 possesses the highest-lying HOMO (−5.665 eV), whereas R2 exhibits the lowest LUMO (ELUMO = −1.005 eV). This ordering already points to R1 as the preferred electron donor and R2 as the preferred electron acceptor in the envisaged transformation. In terms of frontier-orbital matching, the energy difference between EHOMO(R1) and ELUMO(R2) is significantly smaller than the reverse gap |EHOMO(R2) − ELUMO(R1)|, indicating that the most favorable HOMO–LUMO interaction corresponds to a charge transfer from R1 to R2. Consequently, R1 is expected to behave as the nucleophilic partner, while R2 plays the role of an electrophilic substrate in the proposed reaction pathway.

The conceptual-DFT descriptors support this assignment. The chemical potential of R1 (μ = −1.690 eV) is higher than that of R2 (μ = −4.079 eV), confirming that R1 is more prone to donate electron density. In contrast, the global electrophilicity index ω is much larger for R2 (ω = 1.353) than for R1 (ω = 0.180), which clearly identifies R2 as the more electrophilic species. In addition, the nucleophilicity index N is higher for R1 (N = 3.864) than for R2 (N = 2.375), in line with its nucleophilic character. Finally, R2 presents a smaller HOMO–LUMO gap (η = 6.149 eV) compared with R1 (η = 7.949 eV), making R2 globally softer and more reactive, and therefore better able to accommodate the incoming charge from R1. Altogether, these FMO and global reactivity parameters consistently indicate that the preferred interaction in this system is the HOMO of R1 interacting with the LUMO of R2, which is in full agreement with the proposed organic transformation.

**Figure 3:**
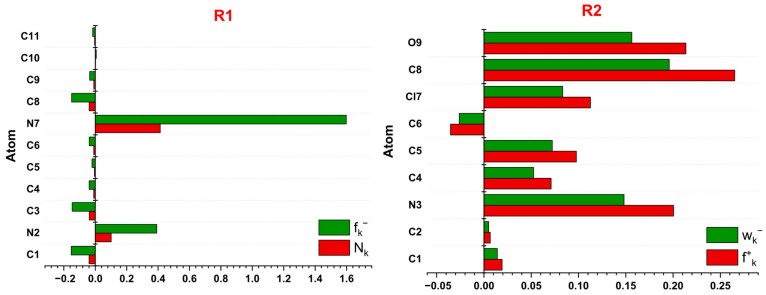
Fukui indices, local electrophilicity and local nucleophilicity of R1 and R2 based on NPA analysis.

Figure 3 presents the local reactivity indices for reagents R1 and R2, namely, the Fukui functions for nucleophilic attack (f_k_^−^) and the local nucleophilicity indices (N_k_) for R1, together with the Fukui functions for electrophilic attack (f_k_^+^) and the local electrophilicity indices (ω_k_) for R2, obtained from DFT/B3LYP/6-31G(d,p) NPA population analysis. For R1, atom N7 clearly exhibits the largest N_k_ and f_k_^−^ values, identifying it as the main nucleophilic center of the molecule and the most favorable site for interaction with an electrophilic partner. In contrast, the local descriptors of R2 highlight the atoms associated with electrophilic behavior: C8 displays the highest f_k_^+^ and one of the largest ωk values, indicating that this carbon is the primary electron-accepting center and thus the most likely target site for nucleophilic attack. N3 and O9 also show relatively high ω_k_ and f_k_^+^ values, pointing to an additional capacity of these sites to stabilize incoming electron density. Overall, the Fukui and local reactivity analyses confirm the global picture derived from FMO and conceptual-DFT descriptors, namely, that R1 behaves as a nucleophilic reagent, with N7 as its dominant nucleophilic site, while R2 acts as an electrophilic partner, with C8 constituting the preferred centers for bond formation in the proposed organic transformation.

### 2.3. Antioxydant Activity

**Figure 4:**
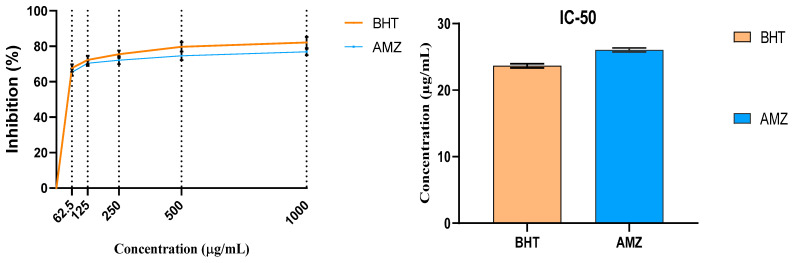
Inhibition percentage and IC-50 values of AMZ relative to BHT, evaluated using the DPPH method. Values are represented as mean ± standard deviations of triplicate experiments.

The DPPH radical scavenging assay reveals (Figure 4 and Table 2) that AMZ exhibits antioxidant activity close to the reference, approaching the efficacy of standard BHT while offering unique advantages. Both compounds exhibit dose-dependent scavenging, with inhibition percentages increasing steadily as a function of concentration (62.5–1000 µg/mL). BHT remains more potent at all doses, achieving 82.10 ± 1.80% inhibition at 1000 µg/mL, compared with 76.88± 3.20% for AMZ. Also, the IC50 values highlight the competitive potential of AMZ. The IC50 of BHT is 23.65 ± 0.22 µg/mL, while that of AMZ is only slightly higher (26.03 ± 0.21 µg/mL), indicating that AMZ needs a slightly higher concentration to neutralize 50% of free radicals. This small discrepancy in IC50 suggests that the molecular structure of AMZ is well optimized for radical scavenging, probably due to efficient electron donor groups or rapid reaction kinetics.

**Figure 5:**
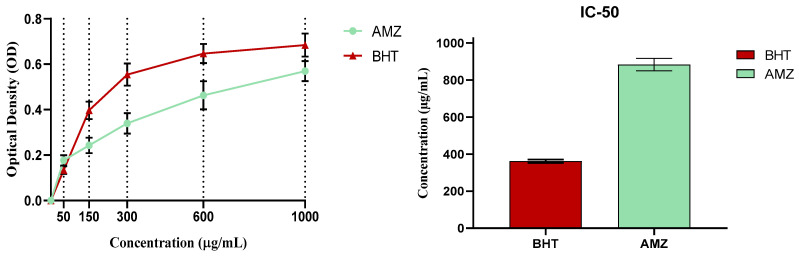
Inhibition percentage and IC-50 values of AMZ relative to BHT, evaluated using the FRAP Method. Values are represented as mean ± standard deviations of triplicate experiments.

The FRAP assay results highlight AMZ as a promising antioxidant candidate with distinct advantages, particularly in low-concentration applications. While the standard antioxidant BHT exhibits superior reducing power at higher concentrations (≥150 µg/mL), AMZ demonstrates notable efficacy at lower doses. At 50 µg/mL, AMZ outperforms BHT with an optical density (OD) of 0.177 ± 0.023 compared to BHT’s 0.134 ± 0.017, suggesting AMZ may be more efficient in scenarios requiring minimal antioxidant concentrations (Figure 5 and Table 3). This low-dose potency could make AMZ advantageous in formulations prioritizing reduced chemical load or lower toxicity, such as cosmetics or sensitive pharmaceutical products.

In the DPPH test shown in Table 4, AMZ (26.03 ± 0.21 µg/mL) displays an antioxidant capacity very close to that of BHT (23.65 ± 0.22 µg/mL). The narrow gap indicates that AMZ performs nearly as efficiently as the commercial reference in scavenging DPPH radicals, confirming that the compound has a competitive free radical-quenching ability. Although AMZ’s IC50 (700.48 ± 22.54 µg/mL) is higher than BHT’s (400.16 ± 8.67 µg/mL), this does not diminish its potential utility. The gradual, dose-dependent increase in AMZ’s reducing power (reaching 0.570 ± 0.044 OD at 1000 µg/mL) reflects a steady and predictable activity profile, which may be preferable for controlled-release systems or applications requiring sustained antioxidant effects. Furthermore, the absence of sharp activity plateaus in AMZ’s curve suggests room for structural optimization—for instance, introducing electron-donating groups like hydroxyl or methoxy substituents, which could enhance its redox capacity and bridge the gap with BHT. The different activity profiles observed in the DPPH and FRAP assays are not unexpected, since these two methods probe distinct antioxidant mechanisms. The DPPH assay primarily reflects the ability of a compound to quench a stable organic radical, whereas the FRAP assay measures ferric ion-reducing power under acidic conditions. Consequently, differences in redox potential, reaction kinetics, medium effects, and structural accessibility may lead to different concentration–response behaviors in the two assays. Thus, the DPPH and FRAP results should be regarded as complementary rather than contradictory.

### 2.4. Antimicrobial Activity of Chloropyridine Derivative

#### 2.4.1. In Vitro Antimicrobial Efficacy of AMZ

**Figure 6:**
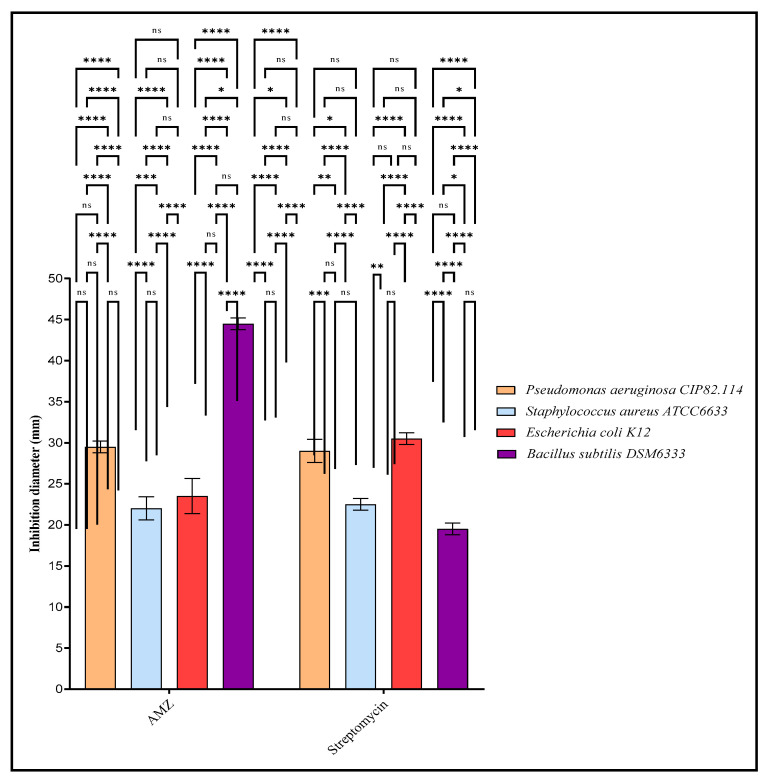
Antimicrobial activity of AMZ against the microbial strains tested. Means (±SD, n = 3) marked with stars indicate significant difference according to two-way ANOVA and Tukey’s multiple interval tests at *p* < 0.05 (* *p* < 0.05, ** *p* < 0.01, *** *p* < 0.001, **** *p* < 0.0001).

In the present study, the disk diffusion assay was used as an initial qualitative screening method to visualize growth inhibition, whereas quantitative antimicrobial potency was further assessed via broth microdilution through determination of MIC and MBC values, as presented in Table 5. Figure 6 highlights the inhibition diameters in mm of AMZ against various microbial strains, comparing its performance with the reference antibiotic, Streptomycin. For *Pseudomonas aeruginosa* CIP82.114, AMZ showed a slightly higher inhibition diameter, 29.5 ± 0.5 mm, compared to Streptomycin, 29 ± 1 mm, indicating comparable antibacterial activity. In the case of *Staphylococcus aureus* ATCC6633, AMZ exhibited a slightly lower inhibition diameter (22 ± 1 mm) compared to Streptomycin (22.5 ± 0.5 mm), suggesting that both compounds demonstrate similar efficacy against this strain. Against *Escherichia coli* K12, AMZ demonstrated a lower inhibition diameter (23.5 ± 1.5 mm) compared to Streptomycin (30.5 ± 0.5 mm), indicating weaker antibacterial activity for AMZ against this strain. For *Bacillus subtilis* DSM6333, AMZ exhibited the highest inhibition diameter (44.5 ± 0.5 mm), significantly surpassing Streptomycin (19.5 ± 0.5 mm), highlighting its superior activity against this strain.

**Figure 7:**
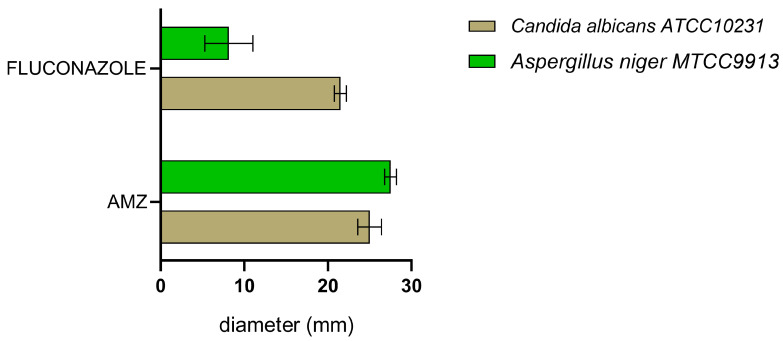
Antifungal activity of AMZ and Fluconazole against *Candida albicans* and *Aspergillus niger*.

Similarly, in fungi, whose activity can be seen in Figure 7, AMZ showed high anti-*Candida albicans* activity with an inhibition of 24 mm, marginally below 25 mm for Fluconazole. AMZ, in contrast, showed even higher efficacy in its inhibition of *Aspergillus niger,* with an inhibition of 30 mm, a considerable improvement over 15 mm for Fluconazole. Overall, AMZ showed high antifungal activity, most prominently against *Aspergillus niger*, with efficacy against *Candida albicans* comparable to that of Fluconazole. Consequently, AMZ was a strong compound, exhibiting high inhibition diameters in both fungal strains.

#### 2.4.2. Minimum Inhibitory Concentration (MIC) and Minimum Bactericidal Concentration (MBC) of New Chloropyridine Derivative

The antimicrobial activity of the compound AMZ was evaluated against a panel of microbial strains, including *P. aeruginosa* CIP82.114, *S. aureus* ATCC6633, *E. coli* K12, *B. subtilis* DSM6333, *C. albicans* ATCC10231, and *Aspergillus niger* MTCC9913, and compared with standard antibiotics, Streptomycin and Fluconazole. The results, summarized in Table 5, demonstrate that AMZ exhibited significant antimicrobial efficacy across all tested strains.

Against bacterial strains, AMZ showed remarkable activity, with MIC and MBC values of 0.008 mg/mL for both *E. coli* K12 and *B. subtilis* DSM6333, indicating strong bactericidal effects. Similarly, against *S. aureus* ATCC6633, AMZ demonstrated an MIC and MBC of 0.016 mg/mL, further underscoring its potency. For *P. aeruginosa* CIP82.114, AMZ exhibited an MIC and MBC of 0.130 mg/mL, which, although higher than the other bacterial strains, still represents considerable activity. In comparison, Streptomycin showed higher MIC and MBC values against these strains, with values ranging from 0.243 mg/mL for *P. aeruginosa* CIP82.114 to 7.125 mg/mL for *B. subtilis* DSM6333, highlighting the superior efficacy of AMZ against these pathogens. The higher susceptibility observed for *Bacillus subtilis* may be related to its Gram-positive cell-envelope architecture, which lacks the outer membrane barrier characteristic of Gram-negative bacteria and may therefore facilitate access of AMZ to membrane-associated or intracellular targets. In the case of *Aspergillus niger*, the stronger activity may reflect differences in fungal membrane and cell-wall organization, as well as possible sensitivity of sterol biosynthesis-related pathways to the chloropyridine diamine scaffold. At this stage, these interpretations should be considered mechanistic hypotheses that may guide future structure–activity studies.

The current results are in strong agreement with previous findings reported by Marinescu and Popa [18], who reviewed a broad range of pyridine derivatives with antimicrobial activity against the same bacterial strains used in this study. Notably, Malhotra et al. [19] synthesized Mannich bases derived from pyridine scaffolds, and several compounds—particularly 12, 15, 16, and 17—showed potent antibacterial activity with MIC values ranging from 6.25 to 12.5 µg/mL against *S. aureus*, *B. subtilis*, *E. coli*, and *P. aeruginosa*. These values are consistent with the results obtained for compound AMZ in the current study, which demonstrated MICs of 0.008 mg/mL (8 µg/mL) for *E. coli* and *B. subtilis*, and 0.016 mg/mL (16 µg/mL) for *S. aureus*. Similarly, Ragab et al. [20] reported on sulfonamide-based pyridine derivatives, compounds 36 and 37, which exhibited MIC values of 5.74 µM (approximately 1.9 µg/mL) against *S. aureus*, 16.12 µM (5.4 µg/mL) against *B. subtilis*, 22.61 µM (7.6 µg/mL) against *E. coli*, and 90.48 µM (30.6 µg/mL) against *P. aeruginosa*. These results are particularly relevant given that AMZ showed a comparable MIC of 0.130 mg/mL against *P. aeruginosa*, a strain that is often resistant to conventional antibiotics.

Furthermore, Brycki et al. [21] synthesized a series of pyridine salts (compounds 51–56), with MIC values ranging from 0.0244 to 6.2485 mM against the tested strains. For example, compound 55 showed strong antibacterial effects with MIC values of 0.0244 mM (6.2 µg/mL) for *S. aureus* and *B. subtilis*, 0.0488 mM (12.4 µg/mL) for *E. coli*, and 0.0976 mM (24.9 µg/mL) for *P. aeruginosa*, placing AMZ among the most potent compounds described to date. These comparisons confirm that compound AMZ exhibits comparable or even superior antibacterial activity relative to several previously reported pyridine derivatives. While our findings align closely with most studies in terms of efficacy against *S. aureus*, *E. coli*, and *B. subtilis*, our MIC value for *P. aeruginosa* (0.130 mg/mL) is slightly higher than the best-performing compounds in the literature, such as compound **6c** (MIC = 0.005 mg/mL) from another study referenced in [22], which suggests that further structural optimization of AMZ could enhance its potency against this more resistant strain.

Regarding antifungal activity, the results obtained for *C. albicans* and *A. niger* are in good agreement with recent reports on pyridine-based scaffolds. Narang et al. [23] synthesized nicotinic acid benzylidene hydrazide derivatives that exhibited antifungal activity against both *C. albicans* and *A. niger*, in some cases comparable to fluconazole, confirming that appropriately substituted pyridine cores can provide potent dual antifungal profiles. More recently, Desai et al. [24] prepared benzimidazole–cyanopyridine–thiazolidinone hybrids (**108a**–**108e**), among which compound **108d** showed MIC values of 50 µg/mL and 25 µg/mL against *C. albicans* MTCC 227 and *A. niger* MTCC 282, respectively—activities that are comparable to, or slightly better than, those of the reference drug ketoconazole (MIC = 50 µg/mL for both strains). In our study, AMZ also demonstrated notable antifungal activity: for *C. albicans* ATCC 10231, the MIC and MBC values were 0.033 mg/mL (33 µg/mL), while for *Aspergillus niger* MTCC 9913, they were 0.030 mg/mL (30 µg/mL). In contrast, fluconazole displayed an MIC and MBC of 0.0156 mg/mL for *C. albicans* ATCC 10231, which is slightly lower than that of AMZ, but for *A. niger* MTCC 9913, fluconazole exhibited a much higher MIC and MBC of 7.125 mg/mL, indicating that AMZ is markedly more effective against this filamentous fungus. Overall, these comparisons position AMZ within the same potency range as other pyridine-based antifungal leads while offering a more balanced activity profile across both *C. albicans* and *A. niger* strains.

Overall, the results indicate that AMZ possesses broad-spectrum antimicrobial activity, with particularly strong efficacy against bacterial strains and notable activity against fungal strains. Its performance is comparable to or better than that of the standard antibiotics Streptomycin and Fluconazole, making it a promising candidate for further development as an antimicrobial agent.

AMZ, as detailed in Table 6, stands out with a molecular weight of 409.40 g/mol and a TPSA of 32.26 Å^2^, both well-suited for oral bioavailability. Its hydrogen-bonding profile (4 acceptors, nON = 4) and molecular flexibility (11 rotatable bonds) provide a balance of solubility and permeability, aligning with foundational drug-likeness principles. Unlike Streptomycin, which suffers from excessive molecular weight (581.57 g/mol) and polarity (TPSA = 336.43 Å^2^), AMZ avoids these pitfalls, ensuring better membrane penetration. Fluconazole (Table 6), though compliant with all drug-likeness rules, has a higher TPSA (81.65 Å^2^) compared to AMZ, validating AMZ’s potential as a competitive oral candidate. BHT (Table 6), while advantageous for permeability due to minimal polarity (TPSA = 20.23 Å^2^), highlights solubility challenges, contrasting AMZ’s balanced physicochemical profile.

Table 7 further supports AMZ’s strong drug-likeness, with compliance under Lipinski, Muegge, Egan, and Ghose rules, ensuring oral absorption and metabolic stability. However, its rejection under Pfizer and GSK rules flags potential ADMET liabilities, such as excessive rotatable bonds (Nroth = 11) or structural complexity, typical of early-stage candidates. Despite this, its acceptance under the Golden Triangle rule underscores its physicochemical equilibrium, a critical marker for reduced toxicity and optimized pharmacokinetics. Fluconazole (Table 7), which meets all criteria, serves as a benchmark for success, while BHT’s rejections (Pfizer/GSK) and Streptomycin’s near-total failures highlight AMZ’s relative promise.

### 2.5. In Silico ADMET Prediction

A potential drug has to exhibit not only low-dose efficacy with low toxicity, but also acceptable pharmacokinetics [25]. These are the attributes that make sure that a drug stays in the body long enough to have an effect and does not build up to be toxic. Thus, the ADMET (absorption, distribution, metabolism, excretion, and toxicity) profile is important to evaluate for a potential drug. The ability to predetermine such properties in silico is a way of shortening the drug development pipeline before actual in vivo or in vitro experiments are initiated. Simulation of ADMET properties can help develop drugs with less later-stage failure [26].

According to the ADMET simulation results (Table 8), AMZ displays a favorable absorption profile, with an intestinal absorption rate of 91.14%. This high percentage suggests efficient uptake through the gastrointestinal tract, supporting the feasibility of oral administration. However, Streptomycin shows 0% intestinal absorption, confirming its well-known limitation regarding oral delivery and highlighting the need for parenteral administration routes such as intravenous injection. Fluconazole exhibits an even higher absorption rate (94.96%), indicating that oral administration is fully suitable for this compound as well.

The VDss for AMZ is 1.55 log L/kg, suggesting moderate tissue distribution for this drug. This indicates that AMZ has good penetrability to a variety of tissues, which is helpful for multi-organ drug treatment. On the other hand, Streptomycin and Fluconazole have negative VDss (−0.351 and −0.441, respectively), indicating that they are less distributed; possibly restricted to the circulation or one of the organs, hence compromising their overall tissue penetration. With respect to BBB crossing, AMZ exhibited a slightly positive value (0.16), revealing its weak capability in permeating the BBB for CNS access. The low BBB penetrability of Streptomycin is −2.031, indicating very poor penetration, which is in accordance with the fact that it is used for systemic infections but not for CNS diseases [27]. Fluconazole, with a permeability of −1.067, is slightly capable of transversing the BBB, but to a much lesser extent than AMZ.

The results for CNS permeability were in agreement with those for BBB. AmZ (log PS −2.22) is not expected to enter CNS; however, Streptomycin (low log PS value of −6.492) demonstrates non-appreciable interaction with the CNS. Fluconazole, with a log PS value of −3.185, has low potential for CNS penetration but may accumulate in the brain at trace amounts.

Metabolic interactions with cytochrome P450 enzymes demonstrate substantial differences between the compounds. AMZ is a common substrate of CYP3A4 and CYP2D6, indicating that it can also be passively metabolized by these two enzymes, with the possible risk of drug–drug interactions [28]. On the other hand, Streptomycin and Fluconazole do not interact with CYP-2D6 or -3A4, which might indicate fewer metabolic interactions. Further, AMZ is also a CYP2D6 and CYP3A4 inhibitor, which might influence the metabolism of other compounds metabolized by these enzymes. Fluconazole CYP1A2 inhibition may interact with other drugs metabolized by the enzyme, and Streptomycin does not inhibit any profiled P450 enzyme. With respect to elimination, AMZ has moderate total clearance (0.96 log mL/min/kg), and its excretion from the body is not slow. The very low clearance of streptomycin (0.005 log mL/min/kg) explains its long presence in the body after administration and may lead to accumulation if used over long periods of time. Fluconazole is a slow-clearance compound, clocking in at 0.29 log mL/min/kg, roughly halfway between AMZ and the very slow EMB. In terms of toxicity, both AMZ and Fluconazole are non-AMES toxic, indicating that they should not be mutagenic and have a low likelihood of causing chronic effects. This renders them safer drugs for further drug development. Unlike this, Streptomycin is AMES toxic (demonstrates mutagenic reactivity and a long-term carcinogenic effect), which may restrict its use, especially in long-duration treatments.

### 2.6. Computational Docking Assessment

**Figure 8:**
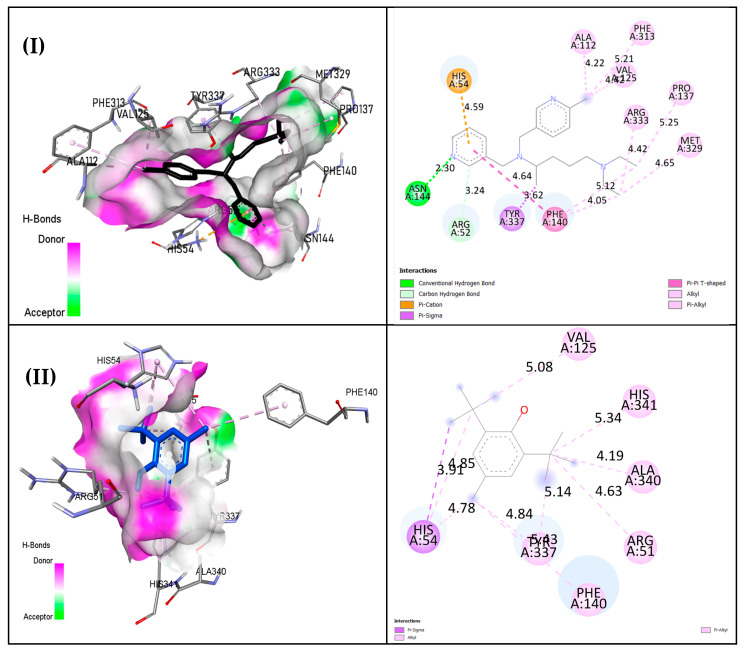
The 2D and 3D docked views of AMZ (**I**) and BHT (**II**) with active site of 2CAG protein.

**Figure 9:**
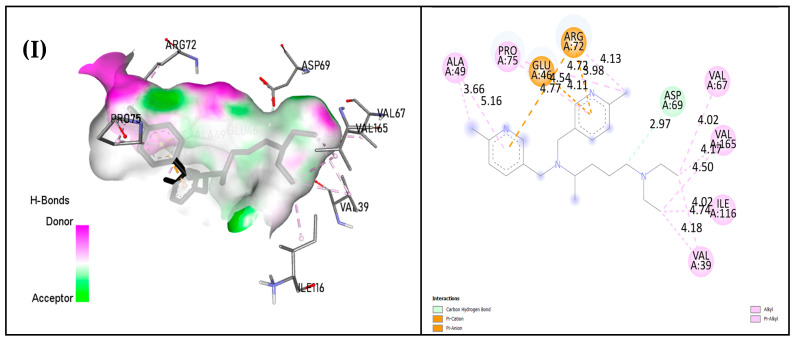

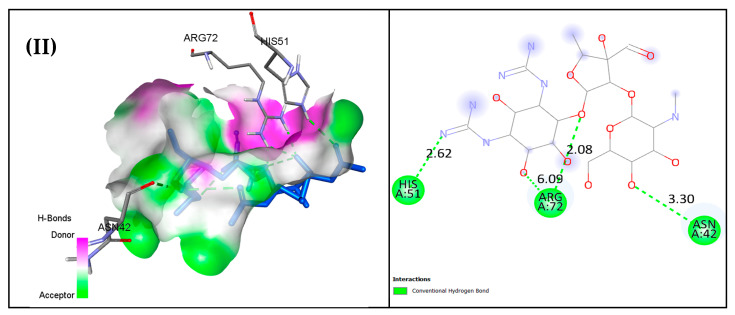
The 2D and 3D docked views of AMZ (**I**) (black color) and Streptomycin (**II**) (blue color) with active site of 3FV5 protein.

A docking study examined the antioxidant, antibacterial and antifungal activities of AMZ by receptor 2CAG, 3FV5 and 1EA1 proteins. The antioxidant action was evaluated in terms of interaction with 2CAG protein, a catalase enzyme essential for the homeostasis of oxidative stress by decomposing hydrogen peroxide [29]. In Table 9, it can be observed that for AMZ, the binding energy was −7.6 kcal/mol, slightly higher than the reference compound BHT (−7.3 kcal/mol). AMZ was involved in hydrophobic interactions with a series of important residues, such as HIS54, PHE313 and TYR337, revealing a stable interaction network binding between AMZ and the receptor (Figure 8). Without hydrogen bonds, a high binding energy and more interaction residues indicate the remarkable potential of AMZ as a convenient antioxidant agent better than BHT. The antibacterial activity of AMZ, as shown in Figure 9, was tested against the 3FV5 protein, a structural template of *E. coli* Topoisomerase IV that is a prerequisite for bacterial DNA replication and bacterial cell division. For AMZ, a binding energy of −5.0 kcal/mol, weaker than Streptomycin (−5.4 kcal/mol), is reported in Table 10, confirming the lower antibacterial affinity of AMZ compared with the reference drug. Streptomycin makes three stability-contributing Hydrogen Bonds (HBs) with HIS51, ARG72, etc., as well. On the other hand, AMZ hydrophobically contacts a larger panel of residues comprising GLU46, ASP69 and ARG72 but not by HB orientation, providing an explanation for its reduced ability to act as an antibacterial agent. While AMZ has partial promise in this regard, streptomycin is a better prospect because of its stronger stabilizing interactions.

**Figure 10:**
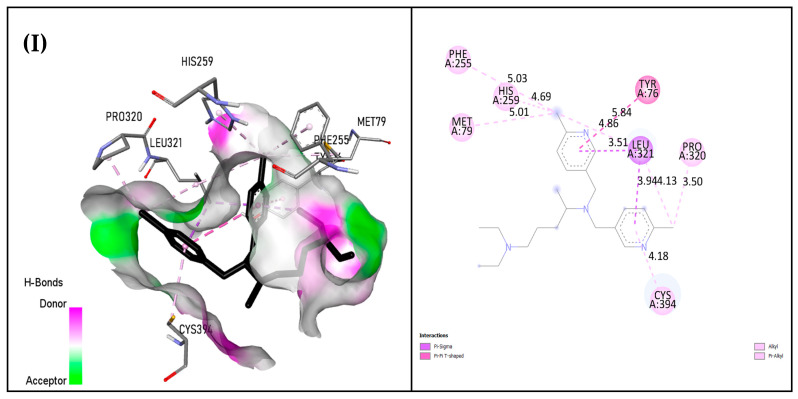

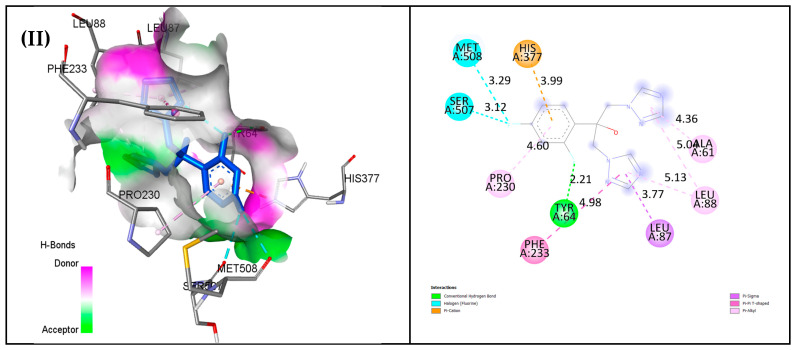
The 2D and 3D docked views of AMZ (**I**) (black color) and Fluconazole (**II**) (blue color) with active site of 1EA1 protein.

The antifungal potential of AMZ was evaluated through molecular docking using the 1EA1 protein (Figure 10), a CYP51-associated target essential for fungal sterol biosynthesis. AMZ exhibited a binding energy of −6.6 kcal/mol, slightly weaker than fluconazole (−7.0 kcal/mol), a widely used antifungal reference (Table 11). Although AMZ shows a less favorable binding affinity, its interaction profile still supports a passive yet relevant inhibitory effect against the fungal pathway. These ligands were further stabilized by hydrophobic interaction with important residues like SER507, MET508 and HIS377, as well as establishing one hydrogen bond each. Although fluconazole is predicted to exert somewhat stronger binding energy, and more interactions were found, like halogen bonding, AMZ could reproduce the binding profile closely, as fluconazole suggests that AMZ can be a good anti-fungal candidate.

The key binding interactions of AMZ involved several residues, depending on the target. In the 2CAG complex, the main contacts included HIS54, ALA112, PHE313, VAL125, ARG333, PRO137, MET329, PHE140, TYR337, ARG52, and ASN144. In the 3FV5 complex, the principal interacting residues were ALA49, PRO75, GLU46, ARG72, ASP69, VAL67, VAL165, ILE116, and VAL39. In the 1EA1 complex, AMZ interacted mainly with SER507, MET508, HIS377, ALA61, LEU88, LEU87, TYR64, PHE233, and PRO320. These interactions support the ability of AMZ to establish stable hydrophobic contacts within the selected binding pockets.

Thus, AMZ has a potent antioxidant capacity, which is even superior to BHT in binding energy and network of interaction. Its antibacterial activity is, however, low compared with streptomycin due to no hydrogen bonding, but it interacts with the key residues in 3FV5. As for anti-fungal activity, AMZ has a binding profile to the substrate that is reminiscent of fluconazole, with slightly less favorable energetics of binding. These results indicate that AMZ could be a potential candidate as an antioxidant and antifungal agent, and further optimization is needed to improve its antibacterial activity.

## 3. Materials and Methods

### 3.1. Items and Materials

Reagents from Merck (Rahway, NJ, USA) were used without any additional purification. The analysis comprised ^13^C NMR and ^1^H NMR studies conducted on a Bruker AM 300 spectrometer (Billerica, MA, USA), functioning at 75.47 MHz for 13C and 300.13 MHz for 1H. CDCl3 and DMSO-d6 served as solvents, with NMR data reported in parts per million (δ), while coupling constants were related to TMS and stated in hertz (Hz). Mass spectrometry was conducted on an Exactive Orbitrap mass spectrometer (Thermo Fisher, Waltham, MA, USA) employing the electron ionization technique (direct sample introduction). The Koffler bank was utilized to ascertain melting points.

### 3.2. Synthesis of N4,N4-Bis((6-chloropyridin-3-yl)methyl)-N1,N1-diethylpentane-1,4-diamine (AMZ)

N1,N1-diethylpentane-1,4-diamine (3 mL, 15.19 mmol) and (2-chloropyridin-4-yl)methanol (4.89 g, 30.38 mmol) were stirred jointly into acetonitrile (20 mL) under 55 C for 8 h, then the solvent was evaporated, washed with diethyl ether, and the same steps were repeated to obtain the final product (light brown solid: 3.84 g, yield: 62%). **1H-NMR** (300.13 MHz, CDCl_3_) = δ 0.98 (6H,m, 2CH_3_), 1.29 (3H,m, CH_3_), 1.41 (2H,m, CH_2_), 1.92 (2H,m, CH_2_), 2.38 (2H,m, CH_2_), 2.44 (1H,m, CH), 2.47 (4H,m, 2CH_2_), 3.69–3.78 (4H, 2CH_2_-pyridyl), 7.65–8.54 (6H_arom_, 3m); **13C-NMR** (75.47 MHz, CDCl_3_): δ (ppm) = 11.8 (2CH_3_); 20.2 (CH_3_); 23.8 (CH_3_); 34.6 (CH_2_); 47.1 (2CH_2_); 48.3 (CH_2_); 52.5 (CH); 53.5 (2CH_2_-N); 123.1 (2CH_arom_); 136.1 (2C_arom_); 138.4 (2CH_arom_); 149.4 (2C_arom_-Cl); 156.2 (2CH-N). mp: 288–291 °C MS (ESI): M+. = 408.18479.

### 3.3. DFT Methodology

This study employed DFT to identify global reactivity descriptors, thereby assessing the electronic properties and reactivity profiles of the examined compounds. These descriptions, derived from the energies of the HOMO and LUMO molecular orbitals, were calculated in the gas phase at the B3LYP/6-31G(d,p) theoretical level, without geometrical restrictions. The acquired values corroborate and substantiate the postulated synthetic routes of the examined compounds. The chemical potential (µ) signifies a molecule’s tendency to interchange electrons with its environment, where lower values indicate more stability against electron transfer processes [30]. Chemical hardness (η), characterized as the energy difference between the highest occupied molecular orbital (HOMO) and the lowest unoccupied molecular orbital (LUMO), quantifies a molecule’s resistance to alterations in electron density; increased hardness indicates enhanced resistance to electronic deformation and, consequently, superior structural stability. The electrophilicity index (ω) measures a species’ ability to take electrons, with higher values indicating a greater capability for electron acceptance in chemical interactions. In contrast, the nucleophilicity index (N), determined in relation to tetracyanoethylene (TCE) as a standard and based on HOMO energy, assesses the electron-donating capacity of compounds, with higher values signifying enhanced nucleophilic character.

All these descriptors are defined according to the following equations [31,32]:

μ=EHOMO+ELUMO2

$$\eta =E_{HOMO}-E_{LUMO}$$

ω=μ22η

$$N=E_{HOMO}(nucleophile)-E_{HOMO}(TCE)$$

Fukui functions for a molecule can be calculated by the following Equations [33]:

$$f_{k}^{+}=P_{k}(N+1)-P_{k}(N)$$

$$f_{k}^{-}=P_{k}(N)-P_{k}(N-1)$$

where P_k_ (N), P_k_ (N − 1), and P_k_ (N + 1) represent the electronic populations of atom k in the neutral, cationic, and anionic forms of the molecule, respectively.

The local electrophilic (ω_k_) and nucleophilic (N_k_) indices at a specific site k in a molecule are calculated using the following expressions [32]:

$$\omega_{k}=\omega \times {f_{k}}^{+}$$

$$N_{k}=N\times {f_{k}}^{-}$$

All DFT calculations were carried out with the inclusion of solvent effects through the IEFPCM implicit solvation model. This continuum treatment was selected to account for the polarization effect of the solvent medium on the electronic structure of AMZ and thereby to improve the connection between the calculated descriptors and the experimentally measured activities in a solution.

### 3.4. Inspection of Antioxidant Activity

#### 3.4.1. Test of Free Radical Scavenging

The antioxidant capacity of the analyzed materials was evaluated using the DPPH radical scavenging technique [34]. A freshly produced methanolic solution of 1,1-diphenyl-2-picrylhydrazyl (DPPH) at a concentration of 0.004% was stored at room temperature in the absence of light to prevent photodegradation. Different quantities of the synthesized chemical (62.5, 125, 250, 500, and 1000 µg/mL) were generated, with butylated hydroxytoluene (BHT) serving as the reference antioxidant. For the assay, 100 µL of each sample concentration was combined with 750 µL of the DPPH solution, and the combination was incubated in the dark for 30 min. The absorbance was subsequently measured at 517 nm utilizing a UV–visible spectrophotometer. A DPPH solution devoid of any added sample functioned as the negative control, whereas 95% methanol was utilized as the blank. The antioxidant activity was quantified as the percentage of DPPH radical inhibition, determined using the following equation:

$$Percent\;Scavenging=\left[\frac{\left(A_{f}-A_{i}\right)}{A_{f}}\right]\times 100$$

where A_f_ is the absorbance of the reaction control, and A_i_ is the absorbance when the test compound is present. By plotting the compound concentrations against their corresponding radical scavenging percentages, the percentage inhibition and IC50 values (the concentration needed to reduce DPPH by 50%) were determined.

#### 3.4.2. Ferric Reducing Power (FRAP) Assay

The antioxidant capacity of the novel synthesized compound was evaluated based on previously reported methods [35]. Using a UV spectrophotometer, absorbance was measured at 700 nm for various doses of the target chloropyridine compound. Butylated hydroxytoluene (BHT) was used as the standard antioxidant. Each sample was tested three times, and the average absorbance was recorded. The ferric-reducing antioxidant capacity was then determined by measuring the substances’ absorbances at 700 nm.

### 3.5. Inspection of Antibacterial Activity

#### 3.5.1. Microbial and Fungal Strains Tested

The antimicrobial potential of the synthesized pyridine derivative was investigated against two fungal species, *Candida albicans* ATCC10231 and *Aspergillus niger* MTCC9913, as well as four bacterial strains, including *Staphylococcus aureus* ATCC6633, *Escherichia coli* K12, *Bacillus subtilis* DSM6333, and *Pseudomonas aeruginosa* CIP82.114. All microbial strains used in this study were supplied by the Laboratory of Biotechnology, Environment, Agri-food and Health at the Faculty of Sciences Dhar El Mahraz, Sidi Mohamed Ben Abdellah University, Fez, Morocco.

#### 3.5.2. Determination of Antibacterial and Antifungal Activity

The antibacterial and antifungal activities of the synthesized pyridine derivative were assessed using the disk diffusion method [36]. Mueller–Hinton (MH) agar plates were used for bacterial cultures, while Malt Extract agar was employed for the growth of *Candida albicans* and *Aspergillus niger*. The tested bacterial and fungal strains were inoculated onto the culture media using the double-layer technique. Fresh bacterial cultures grown in liquid MH medium and fungal cultures cultivated in liquid Malt Extract medium were serially diluted in sterile saline solution (0.9% NaCl) until reaching a turbidity equivalent to 0.5 McFarland standard (approximately 10^6^–10^8^ CFU/mL). Next, 100 µL of each microbial suspension was added to tubes containing 5 mL of soft agar (0.5% agar prepared in MH medium). The inoculated soft agar was then poured onto Petri dishes containing MH agar for bacteria and Malt Extract agar for fungal strains. Sterile Whatman filter paper discs (6 mm in diameter) were impregnated with 20 µL of AMZ solution prepared at a concentration of 100 µg in 100 µL of 5% DMSO and placed on the inoculated agar surfaces containing the bacterial or fungal suspensions (10^6^–10^8^ CFU/mL). For comparison, positive control experiments were carried out under the same conditions using streptomycin (30 µg/disc) as the reference antibiotic for bacteria and fluconazole (15 mg/mL) as the antifungal agent. The inoculated plates were incubated at 37 °C for bacterial strains and 30 °C for fungal strains under dark and humid conditions. The diameters of inhibition zones were measured after 24–48 h of incubation for bacteria and yeast, whereas for *A. niger*, the measurements were taken after 7 days [37]. To minimize diffusion-related bias in agar, all disk diffusion experiments were carried out under standardized conditions, including identical disc diameter, identical sample loading volume, the same vehicle composition, standardized inoculum density (0.5 McFarland), and identical incubation conditions for each tested strain. In addition, the antimicrobial conclusions were not based only on inhibition zone diameters, since MIC/MBC values were also determined via broth microdilution.

#### 3.5.3. Determination of Minimum Inhibitory Concentration (MIC) and Minimum Bactericidal Concentration (MBC)

The minimum inhibitory concentration (MIC) and minimum bactericidal concentration (MBC) of a novel pyridine derivative against four bacterial strains and two fungal strains were determined using the microdilution method outlined by Wiegand et al. [38], so microdilution was conducted by diluting the sample by a factor of 2 in each well, except for the final well, which served as a positive growth control. Following a 24 h incubation period for bacteria, 48 h for *C. albicans*, and 7 days for *A. niger*, the MIC and BMC were ascertained utilizing the colorimetric technique (TTC 0.2% (*w*/*v*)) [39].

All experiments were conducted in triplicate, and the results are expressed as mean values ± standard deviation. Statistical differences among groups were assessed using two-way ANOVA, followed by Tukey’s multiple comparison test. The statistical analyses were carried out using GraphPad Prism 8 software (https://www.graphpad.com/features; accessed on 1 January 2026), with significance considered at α = 0.05 [40].

### 3.6. Drug-Likeness Properties and Physicochemical Features

This study focused on evaluating drug likeness and qualitatively determining the potential of molecules to act as orally administered drugs with good bioavailability [41]. AMZ and biological standards were analyzed for their physicochemical properties and drug-likeness using the Swiss-ADME tool [42]. Parameters, such as molecular weight, the number of heavy atoms, hydrogen bond donors, hydrogen bond acceptors, and rule violations, were assessed. Additional drug-likeness evaluations, including Pfizer Rule 3/75, GSK Rule 4/400, and Pfizer’s Golden Triangle, were performed using the ADMETlab v2.0 platform [43].

### 3.7. Absorption, Distribution, Metabolism, Excretion, Toxicity (ADMET)

The ADMET approach, which is critical for the design and optimization of new drug candidates, is grounded in the work of Lipinski [44], who identified key molecular properties that influence pharmacokinetic behaviors such as absorption, distribution, metabolism, excretion, and toxicity. To predict these ADMET characteristics, the study utilized the online tool pkCSM (http://biosig.unimelb.edu.au/pkcsm/prediction, accessed on 25 November 2025) [45]. Molecular structures in SMILES format were uploaded to the platform to forecast several pharmacokinetic properties, including distribution parameters (e.g., blood–brain barrier permeability, unbound fraction, central nervous system permeability, and steady-state volume of distribution); metabolism (e.g., CYP2D6/CYP3A4 substrate activity and cytochrome P450 inhibition); excretion (e.g., total drug clearance); and toxicity risks (e.g., skin sensitization, hepatotoxicity, and hERG I and II inhibition) [46].

### 3.8. Molecular Docking Analysis

Molecular docking simulations were performed using AutoDock Vina version 1.5.6 [47] to investigate the possible binding interactions between the studied ligands and their biological targets. The protein targets were selected according to two main criteria: (i) their relevance to the biological activities investigated experimentally in this work, and (ii) the availability of crystallographic structures with defined active sites suitable for comparative docking analysis. Accordingly, catalase was selected as an oxidative-stress-related target, topoisomerase IV as an antibacterial target involved in bacterial DNA replication, and a CYP51-based azole-binding model was selected to explore sterol-demethylase-related recognition relevant to antifungal activity. Three protein structures were selected to explore different biological activities. Catalase Compound II (PDB ID: 2CAG) was used to examine the potential antioxidant mechanism, the crystal structure of *E. coli* Topoisomerase IV in complex with an inhibitor (PDB ID: 3FV5) was employed to study antibacterial activity, and the three-dimensional structure of Cytochrome P450 14α-sterol demethylase (CYP51) from *Mycobacterium tuberculosis* bound to fluconazole (PDB ID: 1EA1) was selected to analyze antifungal activity. All protein structures were downloaded in PDB format from the RCSB Protein Data Bank [48], while the reference ligand was obtained from the ZINC database [49,50]. During protein preparation, water molecules and co-crystallized ligands were removed, and Kollman and Gasteiger charges, as well as polar hydrogen atoms, were added to the structures. The ligand molecules were initially drawn and optimized, saved in MOL2 format, and subsequently converted into PDBQT format for docking calculations. The binding regions were defined by generating grid boxes centered on the active sites of each protein with the following coordinates: *E. coli* Topoisomerase IV (x = 12.51, y = −1.13, z = 3.26), Catalase Compound II (x = 59.83, y = 14.86, z = 16.24), and Cytochrome P450 14α-sterol demethylase (x = −18.14, y = −6.64, z = 66.54). The grid box dimensions were fixed at 20 × 20 × 20, with a spacing of 0.375 Å. The predicted ligand–protein binding affinities were expressed in kcal/mol as negative docking scores. Finally, the resulting interactions between ligands and proteins were visualized and analyzed in both two- and three-dimensional representations using BIOVIA Discovery Studio 2019 and PyMOL (The PyMOL Molecular Graphics System, Version 2.0 Schrödinger, LLC, New York, NY, USA). In the present study, docking was used as a comparative and hypothesis-generating tool to explore plausible binding modes of AMZ relative to the reference ligands. Although formal self-redocking of the co-crystallized ligands was not included in this first analysis, the docking calculations were performed using crystallographically defined active sites and identical preparation/scoring conditions for all ligands. This point is acknowledged as a methodological limitation that could be addressed in future work.

## 4. Conclusions

The new chloropyridine derivative AMZ has been efficiently prepared by catalyst-free N-alkylation in acetonitrile, and its structure was unambiguously elucidated by melting point, ^1^H/^13^C NMR and EI–MS data, which are reliable, thereby demonstrating its practical applicability. Functionally, AMZ has a strong antioxidant profile: its DPPH scavenging ability and EC_50_ are highly similar to those of the standard antioxidant BHT, and using FRAP results, we even demonstrated the superiority of AMZ over BHT at lower concentrations, indicating that it can be efficient at low doses yet good for redox.

Meanwhile, AMZ also exhibits broad-spectrum antimicrobial activity and shows the strongest bactericidal activities toward *Bacillus subtilis* and *E. coli* and potent antifungal effects on *Aspergillus niger*, sometimes exceeding streptomycin and fluconazole. In silico predictions (druglikeness, ADMET) indicate that AMZ respects the main rules of an oral drug, presents high predicted absorption in the intestinal region, and is non-AMES toxic. Docking studies allowed us to confirm that its interplay occurs through antioxidant and antifungal-related targets, such as catalase and CYP51. Although molecular dynamics simulations were not included in the present work, such calculations would provide valuable information on the time-dependent stability and flexibility of the docked complexes. This will be considered in future studies to further refine the proposed interaction modes. Collectively, these consistent experimental and theoretical findings suggest AMZ as a potential multi-functional lead and warrant further efforts in structural optimization, enhancement of antibacterial activity (in particular toward *P. aeruginosa*), and in vivo study for therapeutic efficacy and safety.

## Figures and Tables

**Table 1:** Electronic FMO parameters and global reactivity profiles of R1 and R2.

Molecule	HOMO (eV)	LUMO (eV)	η (eV)	N	μ	ω	E_gap_ I	E_gap_ II
R1	−5.665	2.284	7.949	3.864	−1.690	0.180	−5.665	−2.284
R2	−7.153	−1.005	6.149	2.375	−4.079	1.353	----	----

**Table 2:** Free radical scavenging percentage measured for AMZ and standard antioxidant (BHT).

Concentrations (µg/mL)	AMZ	BHT
	%	
62.5	65.39 ± 2.10	67.68 ± 1.90
125	70.43 ± 2.40	72.25 ± 1.50
250	72.14 ± 2.00	75.58 ± 2.20
500	74.61 ± 2.70	79.71 ± 2.40
1000	76.88 ± 3.20	82.10 ± 1.80

Values are represented as mean ± standard deviations of triplicate experiments.

**Table 3:** Capabilities of reducing power measured using AMZ and standard antioxidant (BHT).

Concentrations (µg/mL)	AMZ	BHT
	%	
0	0	0
50	0.177 ± 0.023	0.134 ± 0.017
150	0.243 ± 0.034	0.397 ± 0.038
300	0.340 ± 0.045	0.554 ± 0.049
600	0.463 ± 0.062	0.647 ± 0.042
1000	0.570 ± 0.044	0.684 ± 0.051

Values are represented as mean ± standard deviations of triplicate experiments.

**Table 4:** IC50 calculated using both DPPH and FRAP methods of AMZ and butylated hydroxytoluene.

	BHT	AMZ
DPPH IC-50 (µg/mL)	23.65 ± 0.22	26.03 ± 0.21
FRAP IC-50 (µg/mL)	400.16 ± 8.67	700.48 ± 22.54

Values are represented as mean ± standard deviations of triplicate experiments.

**Table 5:** MIC and MBC of AMZ with standard Streptomycin and Fluconazole against bacterial and fungal strains, expressed in mg/mL.

		*P. aeruginosa* CIP82.114	*S. aureus* ATCC6633	*E. coli* K12	*B. subtilis* DSM6333	*C. albicans* ATCC10231	*Aspergillus niger* MTCC9913
AMZ	MIC	0.130 ± 0.010 ^c^	0.016 ± 0.002 ^b^	0.008 ± 0.002 ^c^	0.008 ± 0.002 ^c^	0.033 ± 0.005 ^c^	0.030 ± 0.002 ^a^
	MBC	0.130 ± 0.021 ^C^	0.016 ± 0.003 ^B^	0.008 ± 0.003 ^C^	0.008 ± 0.003 ^B^	0.033 ± 0.003 ^C^	0.030 ± 0.004 ^a^
Streptomycin	MIC	0.243± 0.045 ^a^	1.56 ± 0.05 ^a^	3.125 ± 0.011 ^b^	7.125 ± 0.024 ^c^	-	-
	MBC	0.243± 0.072 ^A^	1.56 ± 0.07 ^A^	3.125 ± 0.017 ^B^	7.125 ± 0.016 ^C^	-	-
Fluconazole	MIC	-	-	-	-	0.0156 ± 0.007 ^d^	7.125 ± 0.015 ^a^
	MBC	-	-	-	-	0.0156 ± 0.005 ^D^	7.125 ± 0.023 ^A^

Mean values (±SD, n = 3) followed by different small letters (for MIC) and capital letters (for BIC) in the same column are significantly different (two-way ANOVA; Tukey test, *p* < 0.05).

**Table 6:** Physicochemical properties of AMZ and biological standards determined using SwissAdmet.

Compound	MW	TPSA	N-Atoms	nON	nOHNH	Nviolations	Nrotb
**AMZ**	409.40	32.26	27	4	0	0	11
**Streptomycin**	581.57	336.43	40	15	12	0	9
**Fluconazole**	306.27	81.65	22	7	1	0	5
**BHT**	220.35	20.23	15	1	1	0	3

**Table 7:** Drug likeness property of AMZ, Streptomycin and Fluconazole determined using Swiss ADME ^1^ and AdmetLab 2.0 ^2^.

Compound	Drug Likeness							
	Veber ^1^	Muegge ^1^	Egan ^1^	Ghose ^1^	Lipinski ^1^	Pfizer Rule ^2^	GSK Rule ^2^	Golden Triangle ^2^
AMZ	No	Yes	Yes	Yes	Yes	Rejected	Rejected	Accepted
Streptomycin	No	No	No	No	No	Accepted	Rejected	Rejected
BHT	Yes	Yes	Yes	Yes	Yes	Rejected	Rejected	Accepted
Fluconazole	Yes	Yes	Yes	Yes	Yes	Accepted	Accepted	Accepted

**^1^** denotes the drug-likeness parameters obtained from **SwissADME**, whereas superscript **^2^** denotes those obtained from **ADMETlab 2.0**.

**Table 8:** In silico ADMET results of AMZ and standards.

		AMZ	Streptomycin	Fluconazole	Unit
	**Absorption**				
Intestinal absorption (human)		91.14	0	94.964	Numeric (% Absorbed)
	**Distribution**				
VDss (human)		1.55	−0.351	−0.441	Numeric (log L/kg)
BBB permeability		0.16	−2.031	−1.067	Numeric (log BB)
CNS permeability		−2.22	−6.492	−3.185	Numeric (log PS)
	**Metabolism**				
CYP	2D6 substrate	Yes	No	No	Categorical (Yes/No)
	3A4 substrate	Yes	No	No	Categorical (Yes/No)
	2C19 inhibitor	No	No	No	Categorical (Yes/No)
	2C9 inhibitor	No	No	No	Categorical (Yes/No)
	1A2 inhibitior	No	No	Yes	Categorical (Yes/No)
	3A4 inhibitor	Yes	No	No	Categorical (Yes/No)
	2D6 inhibitor	Yes	No	No	Categorical (Yes/No)
	**Excretion**				
	Total Clearance	0.96	0.005	0.29	Numeric (log mL/min/kg)
	**Toxicity**				
	AMES toxicity	No	Yes	No	Categorical (Yes/No)

**Table 9:** Binding energy of AMZ and BHT with 2CAG by using auto dock vina.

Ligand	Binding Energy (kcal/mol)	Ligand–Protein Interactions	Predominant Type of Interaction	Number of Conventional Hydrogen Bond
AMZ	−7.6	HIS54, ALA112, PHE313, VAL125, ARG333, PRO137 MET329, PHE140, TYR337, ARG52, ASN144	Hydrophobic	0
BHT	−7.3	VAL125, HIS341, ALA340, ARG51, PHE140, TYR337, HIS54	Hydrophobic	0

**Table 10:** Binding energy of AMZ and streptomycin with 3FV5 by using auto dock vina.

Ligand	Binding Energy (kcal/mol)	Ligand–Protein Interactions	Predominant Type of Interaction	Number of Conventional Hydrogen Bond
AMZ	−5.0	ALA49, PRO75, GLU46, ARG72, ASP69, VAL67, VAL165, ILE116, VAL39	Hydrophobic	0
Streptomycin	−5.4	HIS51, ARG72, ASN 42		3

**Table 11:** Binding energy of AMZ and Fluconazole with 1EA1 by using auto dock vina.

Ligand	Binding Energy (kcal/mol)	Ligand–Protein Interactions	Predominant Type of Interaction	Number of Conventional Hydrogen Bond
AMZ	−6.6	SER507, MET508, HIS377, ALA61, LEU88, LEU87, TYR64, PHE233, PRO320	Hydrophobic	1
Fluconazole	−7.0	SER507, MET508, HIS377, ALA61, LEU88, LEU87, TYR64, PHE233, PRO230		1

## Data Availability

The original contributions presented in this study are included in the article/Supplementary Material. Further inquiries can be directed to the corresponding author.

## Supplementary Materials

The following supporting information can be downloaded at https://www.mdpi.com/article/10.3390/ijms27062777/s1.
