# Supplementary material for: Design and Biological Profiling of a Drug-like Chloropyridine Diamine as a Dual Antioxidant–Antimicrobial Lead: In Vitro Evaluation and In Silico Multi-Target Studies
Source: Int J Mol Sci. 2026 Mar 19;27(6):2777. doi: 10.3390/ijms27062777 (PMC13027077; doi:10.3390/ijms27062777)
Supplement: Supplementary file 1 [file ijms-27-02777-s001.zip › ijms-4213790-supplementary.pdf]

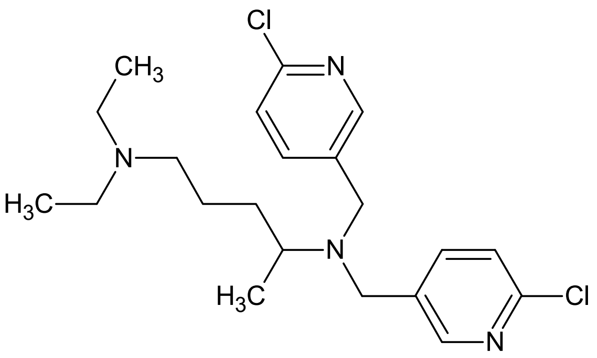

RMN ^{1}H

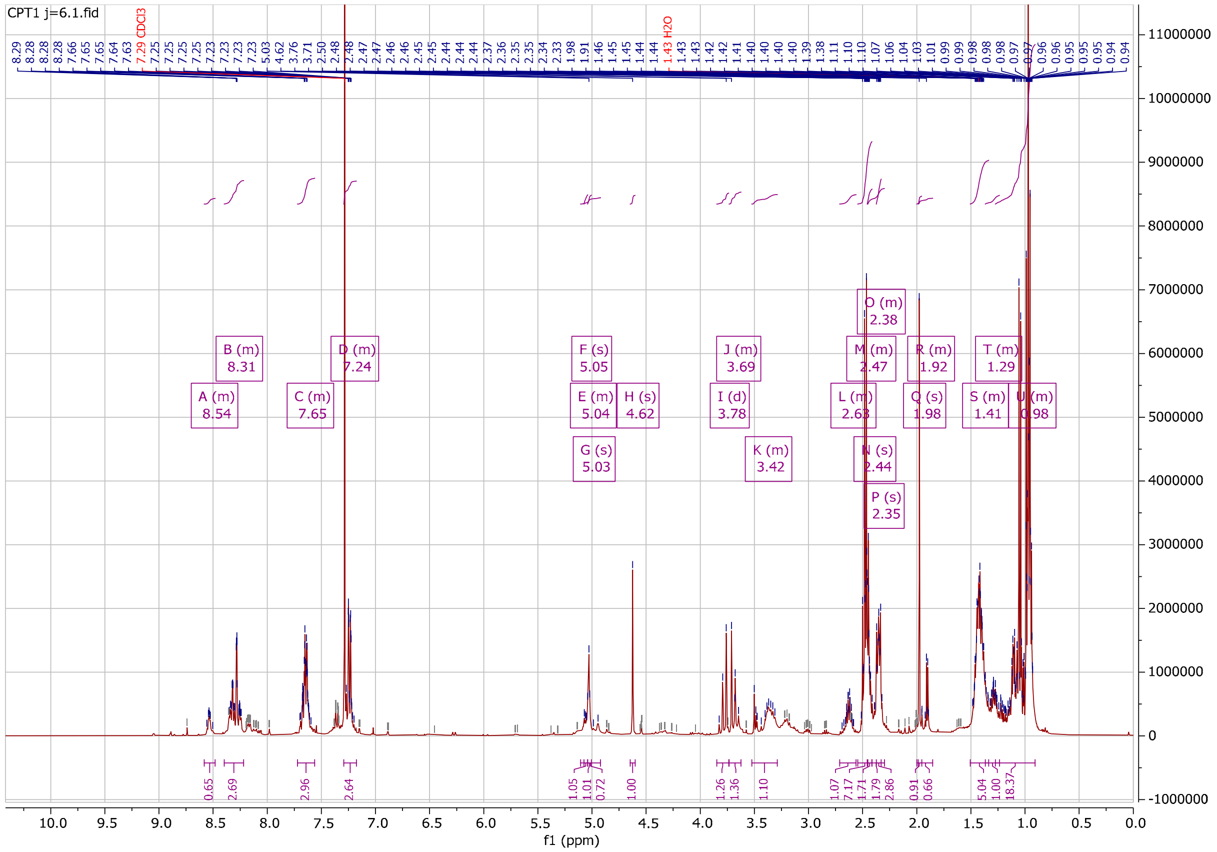

Figure S1: The 1H-NMR spectrum of the compound AMZ

RMN^{13}C

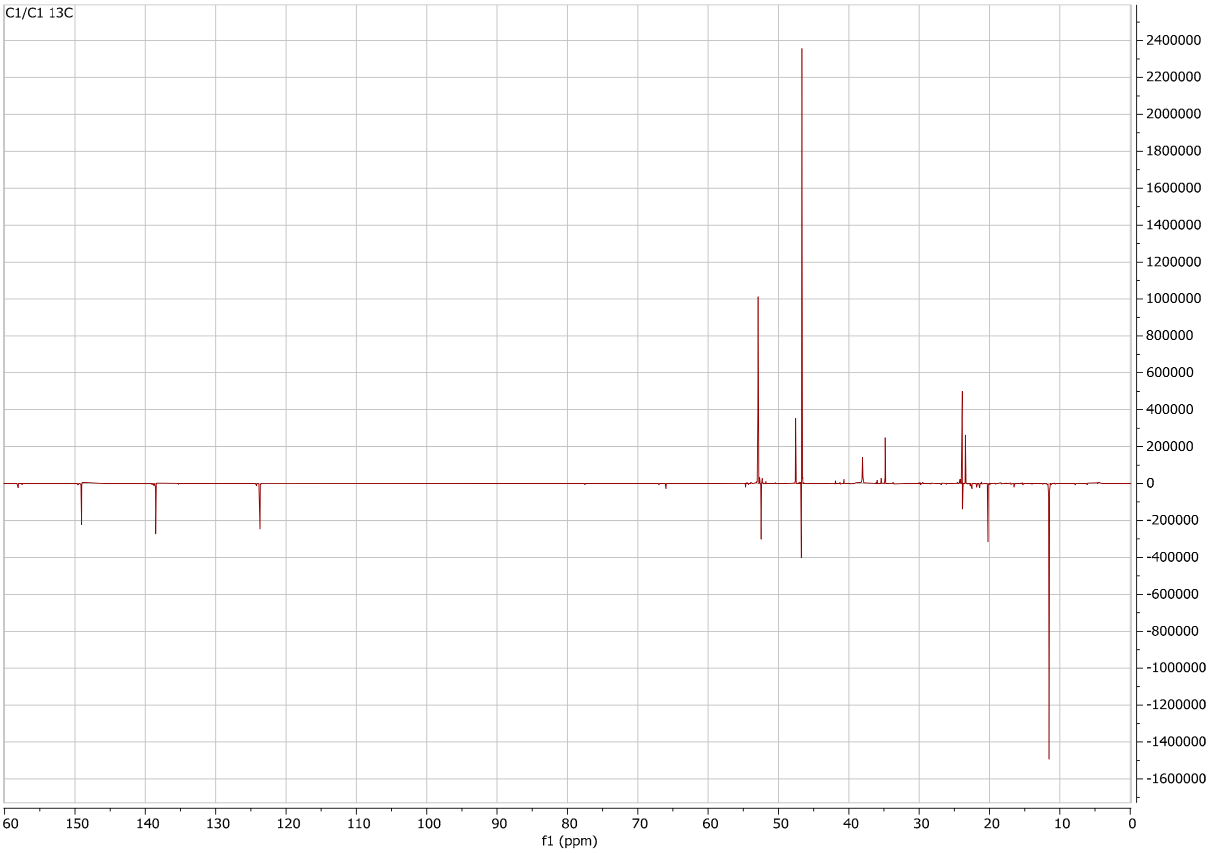

Figure S2: The 13C-NMR spectrum of the compound AMZ;

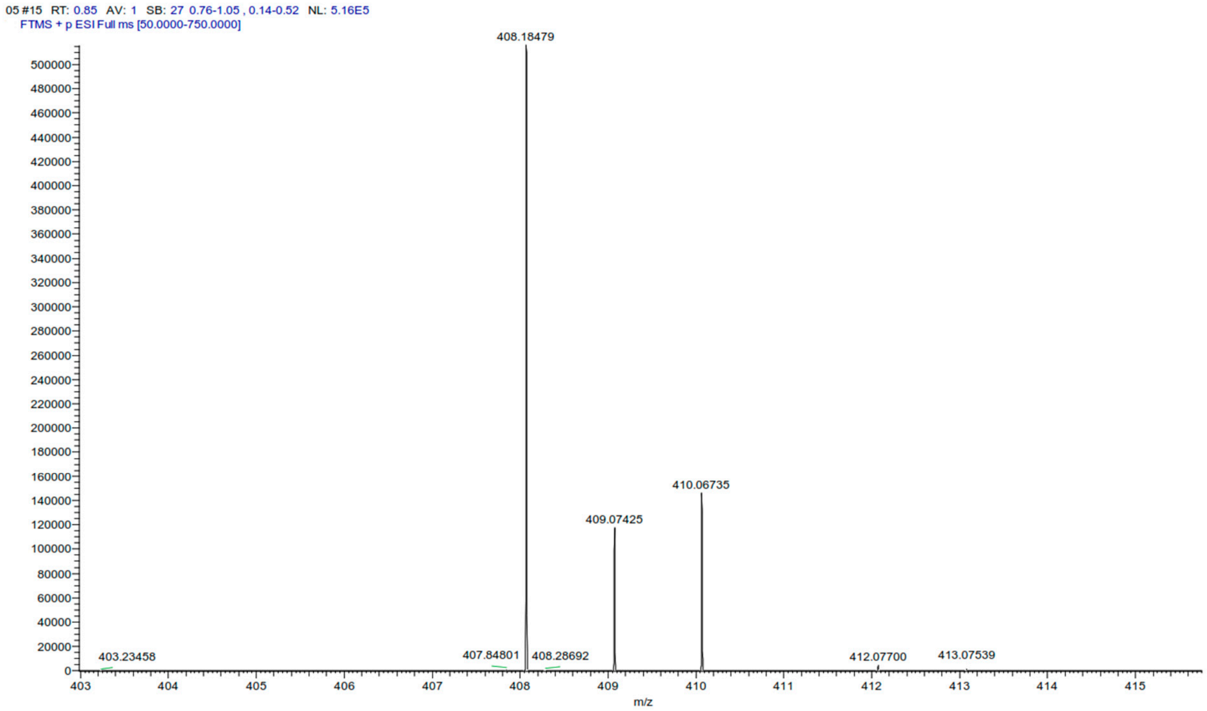

Figure S3: The Masse spectrum of the compounds AMZ.
